# Dyslipidemia and associated factors among adult cardiac patients: a hospital-based comparative cross-sectional study

**DOI:** 10.1186/s40001-024-01802-x

**Published:** 2024-04-15

**Authors:** Alemayehu Abera, Abebaw Worede, Agete Tadewos Hirigo, Rahel Alemayehu, Sintayehu Ambachew

**Affiliations:** 1 Department of Clinical Chemistry, School of Biomedical and Laboratory Sciences, College of Medicine and Health Sciences, University of Gondar, 196, Gondar, Ethiopia; 2 Hawassa University Comprehensive Specialized Hospital, Hawassa, Ethiopia; 3 College of Medicine and Health Science, Faculty of Medicine, School of Medical Laboratory Sciences, Hawassa University, Hawassa, Ethiopia; 4 Pubic Health Institute, College of Medicine and Health Sciences, University of Gondar, Gondar, Ethiopia

**Keywords:** Dyslipidemia, Cardiac patients, Associated factors

## Abstract

**Background:**

Atherosclerotic vascular diseases are a leading global cause of morbidity and mortality. Dyslipidemia, a major modifiable risk factor for cardiovascular disease, remains poorly understood among adult cardiac patients in in the study area. This study aims to determine the prevalence of dyslipidemia and identify associated factors in this population.

**Methods:**

Hospital-based comparative cross-sectional study was conducted from May to August 2021. A total of 319 participants (153 cardiac cases, 166 healthy controls, aged ≥ 18) were included in the study. Socio-demographic, anthropometric, behavioral, and clinical data were collected using the WHO STEPS survey instrument through systematic sampling. Overnight fasting blood samples were obtained, and serum lipid profiles were analyzed using a COBAS 6000 analyzer. Data were analyzed with SPSS version 20.0, employing bivariable and multivariable logistic regression. Statistical significance was set at p < 0.05.

**Results:**

The overall prevalence of dyslipidemia, encompassing at least one lipid abnormality, was 80.3% among 256 participants. Among cardiac cases, the prevalence rates were as follows: 72.5% for low HDL-cholesterol, 12.4% for hypercholesterolemia, 9.8% for elevated LDL-cholesterol, and 30.1% for hypertriglyceridemia. In controls, corresponding rates were 69.9%, 9.6%, 7.2%, and 32.5%. Significant factors linked to low HDL- cholesterol were female gender (AOR: 2.8, 95% CI 1.7–4.7) and obesity (AOR: 2.8, 95% CI 1.1–7.5). Abdominal obesity was associated with hypercholesterolemia (AOR: 5.2, 95% CI 1.9–14.3) and elevated LDL-cholesterol (AOR: 5.1, 95% CI 1.6–15.8). High blood pressure, overweight, and abdominal obesity were significantly linked to hypertriglyceridemia (p < 0.05).

**Conclusion:**

Dyslipidemia was high among the study participants. Overweight, obesity, central adiposity, and high blood pressure were significantly associated with dyslipidemia in cardiac patients. This alarms the need for lipid profile assessment for patients periodically, with treatment follow-up to monitor any rising patterns and cardiovascular-related risks.

## Introduction

Dyslipidemia is a condition that arises as a result of abnormalities in plasma lipids. These abnormalities could be quantitative, qualitative, or both. Quantitatively, dyslipidemia is due to elevated plasma total cholesterol (TC), elevated low-density lipoprotein cholesterol (LDL-c), elevated triglycerides (TG), and reduced high-density lipoprotein cholesterol (HDL-c) levels, occurring singly or in combinations. Qualitatively, dyslipidemia implies changes in the composition of LDL-c, which includes small dense LDL-c, increased TG content, or increased electronegativity of LDL-c [1].

Lipid abnormalities play a central role in the pathogenesis of atherosclerotic cardiovascular disease (ASCVD). Lipoproteins consist of unesterified and esterified cholesterol, phospholipids, triglycerides, and apolipoproteins at variable ratios, densities, and sizes. Their role is to transport lipids in the blood [2, 3]. Based on their buoyant density, lipoproteins are divided into 6 major classes. Chylomicrons, very low-density lipoproteins (VLDL), intermediate-density lipoproteins (IDL), LDL-c and HDL-c, and Lp(a). LDL-c and HDL-c are known as “bad” and “good” cholesterol, respectively. Thus, elevated levels of LDL-c are linked to premature development of atherosclerosis and coronary heart disease; while low levels of HDL-c are associated with an increased risk of ASCVD [2, 4]. HDL-c has anti-oxidative, antithrombotic, and anti-inflammatory properties [5]. VLDL-c and Tg levels are a major determinant of the LDL-c subfraction profile. As plasma TG levels rise, the profile shifts from a predominance of large particles to small dense LDL-c [6]. This is due to an elevated TGs concentration which is associated with higher concentrations of atherogenic small dense LDL-c particles and lower HDL-c concentrations [7].

Hyperlipidemia stands as a robust and firmly established risk factor for cardiovascular disease (CVD). Substantiating this, a Mendelian randomization study reinforces the pivotal causal role of atherogenic lipoproteins, specifically LDL-c, in the development of ASCVD [3]. Hypercholesterolemia is identified as the underlying cause for one-third of ischemic heart diseases (IHD) globally. It is estimated that approximately 2.6 million deaths, constituting 4.5% of the total global mortality, can be attributed to hypercholesterolemia [8]. Within a population, hypercholesterolemia is recognized as the primary factor contributing to the development of atherosclerosis and subsequent coronary heart disease (CHD) [9]. Studies showed that many factors affect the amount of lipid levels in plasma [9, 10]. These could be lifestyle variables, environmental and genetic factors. More commonly, dyslipidemia happens in an acquired condition [11, 12]. For instance, a study conducted by Shephard RJ et al. [13], showed that cigarette smoking bears an independent relationship with triglycerides, HDL-c, and LDL-c.

An epidemiological study by Tesfay F [14], revealed wide disparities in the distribution of main risk factors for CVD such as hypertension, tobacco smoking, alcohol consumption, insufficient intake of fruit and vegetables, obesity, and physically inactivity. These risk factors mainly cause endothelial dysfunction that is associated with the development of inflammation of the arterial wall that damages coronary blood vessels resulting in atherosclerosis [15]. Risk factors: dyslipidemia, hypertension, smoking, obesity, and diabetes mellitus are responsible across the world population for 80% of the CVD burden [16].

The burden of CVD in America, China, Europe, and Africa, remain a significant health challenge. Despite improvements in some areas, factors like obesity and sedentary lifestyles contribute to persistently high CVD prevalence. China faces rising CVD rates due to urbanization. While Europe grapples with healthcare disparities. In Africa, urbanization and lifestyle changes drive a growing CVD burden [17, 18].

LDL-c promotes atherosclerosis through complex inflammatory and immune mechanisms leading to lipid dysregulation. A study has indisputably shown that LDL-c is causal in the atherosclerosis process [19], and the size of the total atherosclerotic plaque burden is determined by both the concentration of circulating LDL-c and by the total duration of exposure to these lipoproteins. As time goes eventually, the enlarging atherosclerotic plaque burden reaches a critical mass beyond which the disruption of a plaque can lead to an overlying thrombus that acutely obstructs blood flow resulting in unstable angina, myocardial infarction, or death [20]. Elevated levels of inflammation-related proteins, including fibrinogen, high-sensitivity C-reactive protein (hsCRP), and interleukin-6 (IL-6), are observed in individuals with atherosclerosis [4].

The escalation of cardiovascular disorders is significantly influenced by the epidemiologic transition, marked by the global trends of increasing urbanization and shifts in lifestyle factors occurring universally [21]. The magnitude of CVD is increasing in low and middle-income countries (LMICs) and causing a big public health catastrophe [22].

Ethiopia is emerging as one of the swiftest-growing economies in Africa, currently experiencing an epidemiologic transition. The rapid urbanization is drawing a substantial population migration from rural to urban areas [14, 23].

The nation has brought about alterations in individuals' lifestyles, encompassing changes in physical activity, nutrition, and behaviors such as smoking, alcohol consumption, and khat use [14].

Lipid-lowering drugs have demonstrated a significant reduction in the risk of myocardial infarction [24], Therefore, the identification of cardiac patients and understanding the pattern of dyslipidemia in this population is crucial for successful management [25]. At least a 50% reduction of LDL-c levels from baseline is recommended to prevent CVD, [26].

Enhanced comprehension of the factors influencing dyslipidemia provides valuable opportunities to implement impactful secondary prevention strategies, leading to a notable reduction in dyslipidemia and, consequently, a decline in ASCVD. However, the prevalence of dyslipidemia appears to be on the rise in numerous developing countries [27]**.** Besides, dyslipidemia in cardiac patients in the Ethiopian population has not been well characterized. Therefore, the main aim of this study was to determine the prevalence of dyslipidemia and associated factors among adult cardiac patients.

## Materials and methods

### Study area, design, and period

This study was conducted at Hawassa University Comprehensive Specialized Hospital, Chronic Diseases Clinic. Hawassa is the capital city of the Sidama region and is located 275 km from Addis Ababa.. The Hospital is one of the biggest hospitals in Sidama regional state that provides health services for more than 12 million people and acts as the referral center for other district hospitals. Hence, facility-based comparative cross-sectional study was conducted from May to August 2021 to assess dyslipidemia and associated factors among cardiac patients.

### Sample size and sampling techniques

The determination of the sample size relied on assessing the difference between proportions, taking into account specific parameters, including a confidence interval (CI) of 95%, a ratio of cardiac patients to healthy controls set at 1:1, and a power of 90%.The sample size was determined, considering the prevalence rates of elevated LDL-c concentrations. Specifically, a prevalence of 14.1% from the National NCDs survey of Ethiopia was considered for the healthy control group [28], while a prevalence of 3.8% reported among patients with cardiac disease in Cameroon was utilized for the exposed group [29].

The sample size for each group was calculated as follows:

$${\mathbf{n}} = \frac{{[{\mathbf{p}}_{{\mathbf{1}}} {\mathbf{q}}_{{\mathbf{1}}} + {\mathbf{p}}_{{\mathbf{2}}} {\mathbf{q}}_{{\mathbf{2}}} \left] {\mkern 1mu} \right[{\mathbf{f}}({{\varvec{\upalpha}}},{{\varvec{\upbeta}}})]}}{{\left[ {{\mathbf{p}}_{{\mathbf{1}}} - {\mkern 1mu} {\mathbf{p}}_{{\mathbf{2}}} } \right]^2}}$$

$$\frac{{\left[ {1.96 + 1.28} \right]^{2} \left[ {\left( {0.038 \times 0.962} \right){\mkern 1mu} + {\mkern 1mu} \left( {0.141 \times 0.859} \right)} \right]}}{{\left[ {0.038 - 0.141} \right]^{2} }} = {\text{156 for each group}}$$

where, = α type I error [level of significance, which is 1–0.95 = 5%], = β type II error [1–0.9 = 10%], [power of the study, 1-β = 90%], Power = the probability of getting a significant result, f[α, β] = 10.5 when the power is 90% and CI of 95%, P_1_ = proportion of dyslipidaemia in cardiac group [0.038], P_2_ = proportion of dyslipidaemia in non-exposed group [0.141], q_1_ = 1-P_1_ (0.962) and q_2_ = 1-P_2_ (0.859). Including a 5% non-response rate, the final sample size was 328 [164 cardiac cases and 164 normal controls]. From the 328 calculated sample sizes, a 97.3% response rate was obtained. Systematic sampling method was carried out to select the study participants.

### Data collection

A structured questionnaire was developed to collect data, customizing the World Health Organization (WHO) STEPS questionnaire to accommodate local needs [26]. Socio-demographic characteristics, factors associated with lifestyle, anthropometric measurements, and clinical parameters/comorbidities were collected using this questionnaire. Socio-demographic and behavioral information was gathered by trained clinical nurses working in the cardiac clinic. Before sample collection, a brief explanation of the study’s aim was provided to all participants.

Height was measured using a stadiometer, and weight was recorded using an electronic digital body balance scale with patients barefoot and wearing light clothes. Waist circumference (WC) was measured at the midpoint between the iliac crest and the lower rib. Men with WC ≥ 102 cm or women with WC ≥ 88 cm were defined as having abdominal obesity. Body mass index (BMI) was calculated as weight divided by height squared (kg/m^2). Underweight, normal weight, overweight, and obese were classified as BMI < 18.5, 18.5–24.9, 25–29.9, and ≥ 30, respectively.

Blood pressure was measured using an analog sphygmomanometer and stethoscope. Elevated blood pressure was considered present when the systolic blood pressure was ≥ 130 mmHg and/or diastolic blood pressure was ≥ 85 mmHg or when there was current use of blood pressure-lowering medication.

### Sample collection & laboratory diagnosis

Laboratory technologists collected blood samples by strictly following laboratory standard operational procedures (SOPs). Approximately 5 ml of whole blood was drawn from each participant using a plain vacutainer tube after an overnight fasting period of 8–12 h. Subsequently, the collected samples were allowed to stand for 30 min to facilitate clot formation. The clotted blood samples were then centrifuged at 4000 rpm for 5 min to obtain serum. In cases where a delay in analysis was expected, serum samples were stored at – 40 ºC until analysis. An enzymatic colorimetric technique was utilized to determine TC and TG levels, while a direct homogeneous method was applied for HDL-c and LDL-c determination using the COBAS 6000 analyzer with c501 (Roche Diagnostics GmbH, Mannheim, Germany).

### Data management and quality control

The questionnaire was prepared in English version and translated to the local Amharic language. The questionnaire was tested at Adare General Hospital which is located 5 km away from the study site with 18 confirmed cardiac patients and the necessary correction was done after the pre-test feedback. Appropriate training was given for data collectors, and socio-demographic, behavioral, and anthropometrics data were collected by two trained nurses under the supervision of the principal investigator. All collected data were checked for consistency and completeness daily by the principal investigator. The pre-analytical, analytical, and post-analytical processes were maintained by following laboratory standards. The proper functioning of the instruments, laboratory reagents, and technical performance was checked daily by using quality control samples before running patient samples as well as along with patient samples. Quality control results were interpreted using Westgard rules and appropriate remedies were taken in case of unacceptable quality control results.

### Data analysis

The collected data were checked for completeness, sorted, coded, and entered into the statistical software Epi-Info version 7.2.1.0, and then it was exported and analyzed by SPSS version 20. The results were presented as frequency and percentage for categorical variables, and means and standard deviation for continuous quantitative variables. Normality of the data was checked graphically via visual plots like the Q-Q plot, the statistical methods like the Kolmogorov–Smirnov normality test, and the Shapiro–Wilk normality test.

The chi-square and independent t-test were used to report the data distribution differences. Pearson’s correlation coefficient was applied to determine the relationship of TC, HDL-c, LDL-c, and TGs with different variables. Bivariate and multivariate logistic regression models were used to assess the statistically significant association between independent and dependent variables. A p-value less than 0.05 was deemed statistically significant with a confidence level of 95%0.3

## Results

### General characteristics of the study participants

A total of 319 [155(48.6%) females and 164(51.4%) males] were enrolled in this study and a 97.8% response rate was obtained. The average age of the study subjects was 44 years, with a standard deviation of 17.8. The age range spanned from 18 to 90 years. Two groups were studied: the first group, 153[64(41.8%) males, 89(58.2%) females] were cardiac patients; while the second group, 166[100 (60.2%) males and 66 (39.8%) females] were non-cardiac patients (controls). Most of the study participants, 58% (n = 188), 30.4% (n = 97), 78.1% (n = 249), and 29.8% (n = 95) were urban dwellers, unable to read and write, married, and housewife, respectively (Table 1).

**Table 1:** Socio-demographic characteristics of the study participants, HUCSH, 2021

Parameters	Category	N (%)
Sex	Male	164 (51.4)
	Female	155 (48.6)
Age in years	Mean ± SD(range)	44 (17.8) (18–90)
Residence	Urban	188 (58.9)
	Rural	131 (41.1)
Educational status	Unable to read & write	97 (30.4)
	Non-formal education	47 (14.7)
	Primary school	50 (15.5)
	Secondary school	58 (18.2)
	Collage & above	67 (21)
Occupation	Housewife	95 (29.8)
	Farmer	61 (19.1)
	Employed	50 (15.7)
	Unemployed	40 (12.5)
	Merchant	15 (4.7)
	Retired	18 (5.6)
	Student	40 (12.5)
Marital status	Single	61 (19.1)
	Married	249 (78.1)
	Separated	3 (0.9)
	Windowed	6 (1.9)
Ethnicity	Oromo	102 (32)
	Amhara	51 (18)
	Tigray	5 (1.6)
	Sidama	120 (37.6)
	Wolayita	7 (2.2)
	Gurage	11 (3.4)
	Kembata	8 (2.5)
	Hadiya	8 (2.5)
	Others	7 (2.2)

### Anthropometric, behavioral, and clinical characteristics of the study participants

The mean (± SD) of SBP, DBP, BMI, and WC of the study subjects were 124.2 (21.9), 76.1 (12.1), 23(5.2), and 91.8 (12.1), respectively. The mean SBP, DBP, and WC were significantly higher in cardiac patients when compared to controls (128.5 vs. 120.1, p = 0.001 for SBP; 77.6 vs. 74.6, p = 0.027 for DBP; 93.3 vs. 90.4, p = 0.029 for WC). In addition, mean BMI (Kg/m^2^) was significantly higher in cardiac patients when compared to controls (24.4 vs.21.6, p < 0.0001). Overall, 17 (5.3%) and 34 (10.7%) of the study subjects had a history of smoking and alcoholism, while 10 (3.1%) and 20 (6.3%) were current smokers and had current alcoholism. Moreover, the history of hypertension, familial history of cardiac disease, and depression was significantly higher in cardiac patients when compared to controls (Table 2).

**Table 2:** Anthropometric, behavioral, and clinical characteristics of the study subjects in HUCSH 2021

Parameters	Category	Total 319 (%)	Cardiac patients 153(%)	Controls 166(%)	p-value
Cardiac duration	Mean ± SD	3.6 (2.9)	3.6 (2.9)	–	–
Treatment duration	Mean ± SD	3.6 (2.8)	3.6 (2.8)	–	–
SBP	Mean ± SD	124.2 (21.9)	128.5(25)	120.1 (17.8)	0.001
DBP	Mean ± SD	76.1 (12.1)	77.6 (13.5)	74.6 (10.6)	0.027
HR	Mean ± SD	81.8 (15.4)	83.4 (17.6)	80.4 (13)	0.10
WC	Mean ± SD	91.8 (12.1)	93.3 (13.6)	90.4 (10.3)	0.029
HPC	Mean ± SD	96.7 (12.8)	98 (13.1)	95.5 (12.5)	0.089
BMI	Mean ± SD	23 (5.2)	24.4 (5.8)	21.6 (4.1)	< 0.0001
Past smoker	Yes	17 (5.3)	10 (6.5)	7 (4.2)	0.34
	No	302 (94.7)	143 (93.5)	159 (95.8)	
Active smoker	Yes	10 (3.1)	5 (3.3)	5 (3)	0.89
	No	309 (96.9)	148 (96.7)	161 (97)	
Current alcohol intake status	Yes	20 (6.3)	11 (7.2)	9 (5.4)	0.51
	No	299 (93.7)	142 (92.8)	157 (94.6)	
Past alcohol intake status	Yes	34 (10.7)	21 (13.7)	13 (7.8)	0.088
	No	285 (89.3)	132 (86.3)	153 (92.2)	
Physical activity	Sedentary	84 (26.3)	46 (30.1)	38 (22.9)	0.18
	Light	159 (49.8)	70 (45.8)	89 (53.6)	
	Moderate	65 (20.4)	34 (22.2)	31 (18.7)	
	Vigorous	11 (3.4)	3 (2)	8 (4.8)	
History of HTN	Yes	66 (20.7)	56 (36.6)	10 (6)	< 0.0001
	No	253 (79.3)	97 (63.4)	156 (94)	
Family history of cardiac disease	Yes	20 (6.3)	16 (10.5)	4 (2.4)	0.003
	No	299 (93.7)	137 (89.5)	162 (97.6)	
History of depressions	Yes	49 (15.4)	46 (30.1)	3 (1.8)	< 0.0001
	No	270 (84.6)	107 (69.9)	163 (98.2)	

*BMI* body mass index, *HR* heart rate, *HPC* hip circumference, *DBP* diastolic blood pressure, *SBP* systolic blood pressure, *WC* waist circumference, *HTN* hypertension

### Pattern of lipid profiles among the study groups

The mean HDL-c was significantly lower in cardiac male patients when compared to cardiac female patients (33.0 vs. 37.6, p < 0.0001). However, the mean HDL-c was insignificantly lower in cardiac male patients when compared to controls (34.3 vs. 35.7, p > 0.05). In addition, mean TC and LDL-c were insignificantly higher in cardiac patients compared with controls (149.9 vs. 147.4, p = 0.61, and 84.8 vs.83.5, p = 0.73) respectively. The mean value of TC was 148.6 mg/dl both in men and women, and the mean value of HDL-c was as low as 32.6 mg/dllin men and 37.6 mg/dl in women, while the mean values of TG and LDL-c were 139.5 mg/dl and 84.1 mg/d in both men and women participants, respectively (Table 3).

**Table 3:** Pattern of lipid profiles among study groups in HUCSH, Ethiopia, 2021

Parameters	Category	Total n = 319	Cardiac patients n = 153	Controls n = 166	p-value
TC	Mean ± SD	148.6 (44.6)	149.9 (45.9)	147.4 (43.4)	0.61
HDL-c	Mean ± SD	35.0 (10.5)	34.3 (10.6)	35.7 (10.2)	0.22
LDL-c	Mean ± SD	84.1 ((32.2)	84.8 (34.1)	83.5 (32.5)	0.73
TG	Mean ± SD	139.5 (70.1)	135.7 (68.8)	143.1 (71.2)	0.34
Non HDL-c	Mean ± SD	113.6 (41.8)	114.2 (44.3)	113 (39.5)	0.81
TC/HDL-c	Mean ± SD	4.3 (1.6)	4.4 (1.7)	4.1 (1.5)	0.43

*TC* total cholesterol, *HDL-c* high-density lipoprotein cholesterol, *LDL-c* low-density lipoprotein cholesterol, *TG* triglyceride

### Characteristics of dyslipidemia, anthropometric and blood pressure

Overall, 256 (80.3%) of the study participants had at least one type of lipid abnormality. This means 126 (39.5%) of the cardiac patients and 130 (40.8%) of the control group had at least one type of lipid abnormality (dyslipidemia).

Over all, dyslipidemia was significantly higher among males 139 (43.6%) when compared to females 117 (36.7%, p = 0.04). However, it was significantly higher in cardiac females when compared to cardiac males (43.8% vs. 38.6%, p = 0.007), respectively. The prevalence of hypercholesterolemia, high LDL-c levels, and low HDL-c levels were insignificantly higher in the cardiac group when compared with the control group. While hypertriglyceridemia and TC/HDL-c > 5 mg/dl were insignificantly higher among the control group compared to the cardiac group. In addition, BMI ≥ 25 kg/m^2^, SBP ≥ 130 mmHg, DBP ≥ 85 mmHg, and WC (≥ 88 for females & ≥ 102 for males) were significantly higher in cardiac patients compared to controls (Table 4). Moreover, overall 101(32%) of the study participants had at least 2 lipid profile abnormalities within a single individual according to NCEP ATP III criteria. Four lipid profile abnormality in a single individual was significantly higher in the cardiac group (11.6%, n = 37) compared to the control group (1.3%, n = 4).

**Table 4:** Characteristics of dyslipidemia, anthropometric variables, and blood pressure of the study subjects in HUCSH, Ethiopia, 2021

Parameters	Category	Total 319 (%)	Cardiac patients 153 (%)	Controls 166 (%)	p-value
BMI (Kg/m^2^)	< 25	225 (70.5)	89 (58.2)	136 (82.4)	< 0.0001
	≥ 25–29.9	59 (18.5)	39 (25.5)	20 (12.1)	
	≥ 30	34 (10.6)	25 (16.3)	9 (5.5)	
WC (cm)	< 88 for females & < 102 for males	192 (60.2)	78 (51)	114 (68.7)	0.001
	≥ 88 for females & ≥ 102 for males	127 (39.8)	75 (49)	52 (31.3)	
SBP (mmHg)	< 130	207 (64.9)	81 (52.9)	126 (75.9)	< 0.0001
	≥ 130	112 (35.1)	72 (47.1)	40 (24.1)	
DBP (mmHg)	< 85	250 (78.4)	110 (71.9)	140 (84.3)	0.001
	≥ 85	69 (21.6)	43 (28.1)	26 (15.7)	
TC (mg/dl)	< 200	284 (89.0)	134 (87.6)	150 (90.4)	0.43
	≥ 200	35 (11)	19 (12.4)	16 (9.6)	
HDL-c (mg/dl)	< 40	227 (71.2)	111 (72.5)	116 (69.9)	0.59
	≥ 40	92 (28.8)	42 (27.5)	50 (30.1)	
LDL-c (mg/dl)	< 130	292 (91.5)	138 (90.2)	154 (92.8)	0.41
	≥ 130	27 (8.5)	15 (9.8)	12 (7.2)	
TG(mg/dl)	< 150	219 (68.7)	107 (69.90	112 (67.5)	0.63
	≥ 150	100 (31.3)	46(30.1)	54 (32.5)	
TC/HDL-c ratio	≤ 5	233 (73)	112 (73.5)	121 (72.6)	0.57
	> 5	86 (27)	41 (26.5)	45 (27.4)	

*BMI* body mass index, *DBP* diastolic blood pressure, *SBP* systolic blood pressure, *WC* waist circumference, *TC* total cholesterol, *HDL-c* high density lipoprotein cholesterol, *LDL-c* low density lipoprotein cholesterol, *TG* triglyceride

### Dyslipidemia and its associated factors

In the bivariate analysis of variables, being female (COR = 2.5; 95% CI 1.2–5.4), having SBP ≥ 130 mmHg (COR = 2.1; 95% CI 1.1–4.3), and having WC ≥ 88 cm for females & ≥ 102 cm for males (COR = 5.2 95% CI 2.4–11.6), were found to be significantly associated with the hypercholesterolemia. However, on the multivariate analysis, performing light to moderate exercise (AOR = 0.18; 95%CI 0.37–0.85) and having WC ≥ 88 cm for females & ≥ 102 cm for males (AOR = 5.2; 95%CI 1.9–14.3) were significantly associated with the hypercholesterolemia.

Besides, being female (COR = 2.6; 95% CI 1.6–4.3) was found to be significantly associated with HDL-c < 40 mg/dl. However, on the multivariate analysis, female (AOR = 2.8; 95%CI 1.7–4.7) and obesity (AOR = 2.8; 95%CI 1.1–7.5) were significantly associated with HDL-c < 40 mg/dl. Moreover, WC ≥ 88 cm for females & ≥ 102 cm for males, and low to moderate physical activity were significantly associated with LDL-c ≥ 130 mg/dl, while high DBP, overweight, and abnormal WC were significantly associated with hypertriglyceridemia (Table 5).

**Table 5:** Factors associated with abnormal levels of lipid profiles according to NCEP ATP III in HUCSH, Ethiopia, 2021

Explanatory variable	At 95%CI	Outcome variables			
		TC ≥ 200 mg/dl	HDL-c < 40 mg/dl	LDL-c ≥ 130 mg/dl	TGs ≥ 150 mg/dl
Sex = female:	COR AOR	2.5 (1.2–5.4)^*^	2.6 (1.6–4.3)^***^	3.3 (1.4–8.1)^**^	0.81 (0.5–1.3)
		0.93 (0.37–2.3)	2.8 (1.7–4.7)^***^	1.6 (0.57–4.4)	_
Age: ≥ 45 years	COR AOR	1.7 (0.89–3.6)^§^	0.76 (0.47–1.2)	1.2 (0.57–2.7)	1.0 (0.64–1.6)
		0.81 (0.35–1.8)	–	_	_
Residence: urban	COR AOR	1.2 (0.58–2.5)	0.77 (0.47–1.2)	1.2 (0.53–2.7)	1.1 (0.7–1.8)
		_	_	_	_
Alcoholism: yes	COR	0.89 (0.2–4)	1.3 (0.52–3.5)	1.2 (0.27–5.5)	0.71 (0.25–2.0)
		_	_	_	_
Past smoking: yes	COR AOR	0.48 (0.11–2.1)	1.4 (0.66–2.9)	0.65 (0.15–2.9)	2.3 (0.92–5.7) §
		_	_	–	1.7 (0.57–4.9)
Lifestyle sedentary:	COR AOR	0.2 (0.04–0.98)*	1 (0.24–4.1)	0.11 (0.02–0.51)^**^	0.6 (0.17–2.1)
		0.12 (0.01–0.67)^*^	_	0.06 (0.01–0.3)^**^	_
Low to modest exercise:	COR AOR	0.35 (0.09–1.4) §	1.1 (0.3–4.3)	0.15 (0.04–0.57)^**^	0.51 (0.15–1.7)
		0.18 (0.37–0.85)*	_	0.07 (0.01–0.3)**	_
FHH: yes	COR AOR	1.4 (0.612–3.1)	1 (0.55–1.8)	1.3 (0.56–3.4)	1.0 (0.57–1.8)
		_	_	_	_
FHHD: yes	COR AOR	1.5 (0.41–5.3)	1.7 (0.54–5.1)	0.55 (0.07–4.3)	1.2 (0.46–3.1)
		_	_	_	_
Depression: yes	COR AOR	1.2 (0.45–2.9)	1.3 (0.64–2.6)	0.95 (0.31–2.9)	0.44 (0.2–0.95)*
		_	_	_	0.31 (0.13–0.7)
SBP: ≥ 130 mmHg	COR AOR	2.1 (1.1–4.3)*	0.83 (0.51–1.4)	1.8 (0.82–4) §	1.4 (0.83–2.2)
		1.4 (0.59–3.6)	_	0.99 (0.35–2.8)	_
DBP: ≥ 85 mmHg	COR AOR	2.1 (0.97–4.4) §	1.1 (0.6–2)	2.3 (1.0–5.3)*	2.1 (1.2–3.7)**
		1.4 (0.55–3.6)	_	1.9 (0.68–5.8)	1.9 (1.1–3.5)*
BMI:25–29.9 kg/m^2^	COR AOR	1.7 (0.76–4.1) §	1.6 (0.82–3.3) §	1.1 (0.38–3.0)	2.1 (1.1–3.8)*
		1.5 (0.46–5.0)	1.5 (0.7–3.3)	_	2.0 (1.1–3.8)*
BMI: ≥ 30 kg/m^2^	COR AOR	1.7 (0.59–4.8)	0.68 (0.32–1.4)	1.5 (0.49–4.8)	2.5 (1.2–5.2)*
		1.6 (0.44–6.1)	2.8 (1.1–7.5)*	_	1.6 (0.7–3.7)
WC: ≥ 88 cm for females & ≥ 102 cm for males	COR AOR	5.2 (2.4–11.6)***	0.76 (0.46–1.2)	4.9 (2.0–12.0)***	2.3 (1.4–3.8)**
		5.2 (1.9–14.3)**	_	5.1 (1.6–15.8)**	2.0 (1.2–3.5)**

Reference category: male, age < 45 years, rural, no alcoholism, no smoking, vigorous exercise, no familial history of hypertension, no familial history of heart disease, no depression, SBP < 130 mmHg, DBP < 85 mmHg, BMI < 25 kg/m^2^, normal WC(≤ 88 cm in females and ≤ 102 cm in males)

*BMI* body mass index, *DBP* diastolic blood pressure, *FHH* familial history of hypertension, *FHHD* familial history of heart disease, *SBP* systolic blood pressure, *WC* waist circumference, *TC* total cholesterol, *HDL-c* high density lipoprotein cholesterol, *LDL-c* low density lipoprotein cholesterol, *TG* triglyceride

*p < 0.05, **p < 0.01, ***p < 0.001

### Correlation of dyslipidemia in relation to different variables among cardiac patients

Pearson correlation analysis showed that TC, LDL-c, HDL-c, and TG levels were inversely correlated with duration since the diagnosis of cardiac disease and treatment started. In addition, DPB and WC were positively significantly correlated with TC, LDL-c, and TG.

Pearson’s correlation analysis showed that both serum TC and LDL-c levels were significantly correlated with heart rate (r = 0.275, p < 0.001 for TC) (r = 0.265, p < 0.001 for LDL-c) while serum TG levels were significantly correlated with both BMI and HPC (r = 0.312, p < 0.0001 for BMI) (r = 0.333, p < 0.0001 for HPC), respectively.

Serum TC, LDL-c, and TG levels were significantly correlated with both WC and DBP [(r = 0.167, p = 0.04), (r = 0.163, p < 0.04), (r = 0.355, p < 0.0001) for WC]; [(r = 0.192, p = 0.02),( r = 0.169, p = 0.04), (r = 0.236, p = 0.003) for DPB] respectively(Table 6).

**Table 6:** Correlation of dyslipidemia with independent variables among cardiac patients in HUCSH 2021

Variables	Mean ± SD	Significance	TC	HDL-c	LDL-c	TG
Cardiac duration	3.6 (± 2.9)	Correlation coefficient (r)	− 0.263	− 0.095	− 0.215	− 0.135
		p-value	0.003	0.24	0.008	0.09
Treatment duration	3.5 (± 2.8)	Correlation coefficient (r)	− 0.234	− 0.108	− 0.214	− 0.124
		p-value	0.004	0.18	0.008	0.12
DBP	77.6 (± 13.5)	Correlation coefficient (r)	0.192	-0.38	0.169	0.236
		p-value	0.02	0.64	0.04	0.003
Heart rate	83.3 (± 17.6)	Correlation coefficient (r)	0.275	0.021	0.265	0.055
		p-value	0.001	0.79	0.001	0.50
Hip circumference	98 (± 13.1)	Correlation coefficient (r)	0.148	− 0.084	0.12	0.333
		p-value	0.07	0.30	0.14	< 0.001
BMI	24.4 (± 5.8)	Correlation coefficient (r)	0.151	− 0.109	0.154	0.312
		p-value	0.06	0.18	0.06	< 0.001
WC	93.3 (± 13.6)	Correlation coefficient (r)	0.167	− 0.149	0.163	0.355
		p-value	0.04	0.07	0.04	< 0.001

## Discussion

Dyslipidemia is one of the important risk factors for CVD. This study indicated that overall 80.3% (95% CI 75.9–84.6) of the participants had at least one type of lipid abnormality. The most frequently encountered lipid abnormality was a low level of HDL-c (71.2%), followed by hypertriglyceridemia (31.3%). The result of dyslipidemia in the current study was in line with the study conducted in Egypt among CAD patients (80%) [30]. However, this finding was lower than the study conducted in Iran (85.4%) [31]. On the contrary, the finding of this study was higher than other studies conducted in Cameroon (26%) [29] and in Ethiopia (66.7%) [32]. The variation in reports might be due to differences in lifestyles such as unhealthy diet and physical inactivity, mental stress due to economic, social, and cultural factors, coronary-prone behavior, genetics, proinflammatory conditions, and diagnostic criteria or differences in cutoff values.

Data from several studies showed that low HDL-c is not uncommon in the general population [33–35]. In the current study, the prevalence of a low level of HDL-c was 71.2%. Similarly, low levels of HDL-c occurred in Ethiopia among 68.7% of study participants in a community-based survey [28]. However, the current result was high compared to a study conducted among Peru Migrants (36.5%) [36]. Low levels of HDL-c are a strong predictor of the occurrence and reoccurrence of myocardial infarction and stroke [37, 38]. This study found that compared with cardiac females, cardiac males had a higher level of low HDL-c (87.5% vs. 61.8%, p < 0.0001). The finding of this study agrees with the Egyptian study [30]. Contrary to this study, in a community-based survey of Ethiopia, low levels of HDL-c in females (73.5%) had been found compared to males (64.8%) [28]. Additionally, the finding was not in line with Cameroon [29] and Iran studies [31] in which elevated low levels of HDL-c were found in females. The variation could be genetic factors between populations and age differences between the studies. In addition, obesity and cigarette smoking were higher in men than females. HDL-c exerts its cardio-protective effects mainly via its role in the reverse cholesterol transport pathway by promoting the removal of cholesterol from peripheral cells and preventing atherosclerosis [39]. More recent data have confirmed that individuals with lower HDL-c levels (< 30.1 mg/dl) were independently associated with a higher risk of CVD, cancer, and other mortality compared with individuals in the reference ranges of HDL-c levels [40].

In this study, the prevalence of raised TG was 30.1%. This finding was almost comparable with the report from Malaysia among CHD patients (31%) [41] and Iran (33.9%) [31], whereas it was higher than the study conducted in Cameroon (18.9%) [29]. However, this finding was lower than the study conducted in Egypt (45%) [30], Pakistan (66%) [42], and India (63%) [43]. These differences might be due to differences in physical inactivity, genetic factors, diet, and lifestyle of the study participants. Triglyceride concentration has strong associations with ASCVD risk [44]. Atherogenic dyslipidemia may play a central role in endothelial dysfunction, which is important for the pathogenesis of atherosclerosis, insulin resistance, as well hypertension. The clinical benefit of both triglycerides and LDL-c lowering might be related to the absolute reduction in VLDL and LDL particles, respectively [45].

In this study, TC and LDL-c were inversely and significantly correlated with the duration of cardiac treatment. This might be due to the therapeutic approach which was used to lower blood pressure, dietary modification, cessation of smoking, and alcoholism and have an impact to reduce the risk of dyslipidemia. Modifying risk factors could potentially reduce the risk of myocardial infarction by more than 80% to 90% [46].

The prevalence of hypercholesterolemia (12.4%) and raised LDL-c (9.8%) found in this study were lower than the studies conducted among CVD patients in Iran (38.0% and 32.3%) [31], and CHD patients in Malaysia (51% and 15.5%) [41], CHD patients in Egypt (59% and 55%) [30], and Pakistan (39.3% and 39.7%) [47]. However, it was lower than the Cameroon study (3.4% and 3.8%) [29]. The observed difference may be due to the difference in socio-demographics, individual lifestyle, genetic variation, study design, and sample size.

The LDL-c/HDL-c ratio might be a more important risk factor than LDL-c itself in CAD patients [48]. LDL-c/HDL-c ratio predicts cardiovascular events and the progression of coronary artery disease [48, 49]. Nicholls et al. have demonstrated that LDL/HDL-cholesterol ratio > 2.0 was related to plaque progression while that < 1.5 was significantly related to plaque regression [49]. The study revealed that 27% of cardiac patients had elevated LDL-c/HDL-c ratios, which make them vulnerable to an increased risk of CHD major adverse cardiovascular events.

The present study depicted that TC and LDL-c were significantly correlated with heart rate and DBP in cardiac patients. As the age goes older, there is a natural tendency for the blood pressure to rise, which could be because of endothelial atherosclerotic changes which increase the stiffness of the arteries in the blood vessel [50].

In this study, low serum HDL-c was significantly associated with the female sex. A variety of data suggest that premenopausal women have a higher HDL-c level than do menopause [51, 52]. Which noted an adverse effect on serum lipid levels in postmenopausal women (> 50 years) occurred due to decreased levels of estrogen production after menopause [53]. Estrogens significantly inhibit LDL transcytosis by down-regulating the expression of endothelial scavenger receptor class B type I [54]. This explains why physiological levels of estrogen reduce LDL transcytosis in arterial endothelial cells of females. This finding offers one explanation why women have a lower risk than men of ASCVD before menopause [55].

In this study, hypertriglyceridemia was significantly associated with DBP. Patients with DBP ≥ 85 mmHg were 1.9 times more likely to develop hypertriglyceridemia compared with normal DBP. This finding was similar to one study in young Egyptian hypertensive CAD patients [30]. The prevalence of DBP was higher in cardiac patients compared to controls (28.1% vs. 15.7%, p = 0.001). This was consistent with a study conducted on CVD patients in Iran [31] and CHD patients of Spain [56]. Hypertriglyceridemia can lead to the development of atherosclerosis by several mechanisms. Low HDL-C levels are commonly escorted by elevated TG levels, and the combination appears to be the most severe combination for hastening vascular damage. The formation of small dense LDL is favored when plasma TG levels exceed 150 mg/dl [57], due to VLDL overproduction resulting in increased exchange of HDL cholesteryl esters for TGs. Cholesteryl ester depletes small dense LDL-c particles that are taken up by arterial wall macrophages causing atherosclerosis. Triglyceride-rich lipoproteins not only augment endothelial dysfunction but also facilitate monocyte infiltration into the arterial wall, and triglyceride-enriched HDL particles exhibit reduced cholesterol efflux capacity [7, 58].

Obesity was found in 16.3% of cardiac patients. This was lower than CHD patients in Saudi Arabia (58.8%) [59], Pakistan (45.2%) [42], and the CVD patients of Cameroon (27.9%) [29]. Overweight and obesity combined were more prevalent in cardiac patients than in the control group (41.8% in cardiac and 18.2% in the control group; p < 0.0001). The observed overweight and/or obesity might be due to a lack of physical activity, lifestyle changes, and dietary patterns such as a higher intake of fat and reduced fiber intake. The rapid development of obesity will undoubtedly accelerate the prevalence of dyslipidemia. People who were obese have been linked to many different health conditions, including CVD, arterial hypertension, type 2 diabetes mellitus, some forms of cancer, and abnormalities of liver function [60, 61].

Cardiac patients who were overweight [AOR = 2; 95% CI (1.10–3.8); p < 0.05)], abdominal obesity [AOR = 2.0; 95% CI (1.2–3.5), p < 0.01], were 2 times more likely to develop hypertriglyceridemia than their counterparts. Pearson correlation analysis also showed that both BMI and WC positively correlated with triglyceride concentration (r = 0.312, p < 0.0001 for BMI) and (r = 0.355 p < 0.0001 for WC). This indicated that the risk of a raised level of TG consistently increased with BMI (25–29.9 kg/dl) and WC. Females had a significantly higher rate of abdominal obesity and overweight when compared to males (62.9% vs. 29.7%, p < 0.0001) and (33.7% vs. 14.1%, p = 0.01), respectively. Overweight and abdominal obesity are independent risk factors for CVD [62]. The development of obesity leads to insulin resistance and it is a major underlying risk factor in the pathogenesis of both dyslipidemia and hypertension [60, 62].

The present study showed that a low level of serum HDL-c was significantly associated with obesity [AOR = 2.8; 95% CI (1.1–7.5); p < 0.05]. After adjusting for other potential confounding variables, the odds ratio for low HDL-c among obese individuals was increased by 2.8 times the risk of developing a low level of HDL-c. The dyslipidemia of insulin resistance is characterized by elevated levels of TGs, low HDL-c, and small dense LDL particles [62]. Obesity is an important independent risk factor for low HDL-cholesterol, suggesting that this could add to other existing forces responsible for the rising burden of cardiovascular risk factors.

In the current study, increased WC was an independent predictor of hypercholesterolemia and LDL-c [AOR = 5.2; 95% CI (1.9–14.3); p < 0.01] [AOR = 5.1; 95% CI (1.6–15.8); p < 0.01], respectively. Females had a significantly higher rate of abdominal obesity compared to males (62.9% vs. 29.7%, p < 0.0001). An increased WC is associated with more subcutaneous fat. Visceral fat is metabolically active; the oxidative process perpetuates an inflammatory response in the subendothelial space, as activated cells secrete pro-inflammatory molecules, including free fatty acids, adiponectin, and adipocytokines such as TNF-α and IL-6, which cause pro-inflammatory damage and reactive oxygen species production resulting in disease states like hypertension [63]. High levels of free fatty acids and inflammatory cytokines contribute to the alteration of the signaling to insulin resistance that is present in many patients with overweight or obesity. Insulin resistance and atherosclerosis are also strongly linked to excess visceral adipose tissue [64].

In this study, TC and LDL-c dyslipidemia were inversely and significantly associated with a sedentary lifestyle [AOR = 0.12; 95% CI (0.01–0.67); p < 0.05; AOR = 0.06; 95% CI (0.01–0.3); p < 0.01], respectively. A sedentary lifestyle came up with the protective effect in this study. This might be due to the reluctance to provide the correct information, the nature of the data that relies on self-reporting, which is highly prone to response bias. Other possible reasons may be a smaller number of participants in every category which may hamper the statistical analysis. A low level of physical activity has been noted to be a significant risk factor for CHD. The protective effect of physical activity was reported in the INTERHEART study [65].

In conclusion, regardless of the study participants, the overall prevalence of dyslipidemia in the current study was high. The most frequently encountered lipid abnormality was a low level of HDL-c followed by hypertriglyceridemia. There was no observed statistical difference in most forms of lipid abnormality between the cardiac and healthy control groups. Obesity, overweight, abdominal obesity, increased DBP, and being female were significantly associated with an increased risk of dyslipidemia.

Hence, it is essential to regularly screen for and manage lipid abnormalities to enhance the overall health and well-being of the patient. Besides, health education should be provided to improve awareness about lipid abnormalities and associated risk factors for preventive purposes.

## Data Availability

The data is available on the primary and corresponding author up on request.
